# Somatostatin Analogues according to Ki67 index in neuroendocrine tumours: an observational retrospective-prospective analysis from real life

**DOI:** 10.18632/oncotarget.6686

**Published:** 2015-12-19

**Authors:** Antongiulio Faggiano, Anna Chiara Carratù, Elia Guadagno, Salvatore Tafuto, Fabiana Tatangelo, Ferdinando Riccardi, Carmela Mocerino, Giovannella Palmieri, Vincenzo Damiano, Roberta Siciliano, Silvana Leo, Annamaria Mauro, Lucia Franca Tozzi, Claudia Battista, Gaetano De Rosa, Annamaria Colao

**Affiliations:** ^1^ Thyroid and Parathyroid Surgery Unit, Istituto Nazionale per lo Studio e la Cura dei Tumouri “Fondazione G. Pascale” IRCCS, Napoli, Italy; ^2^ Endocrinology Unit, Department of Clinical Medicine and Surgery, Università di Napoli Federico II, Napoli, Italy; ^3^ Pathology Unit, Department of Advanced Biomedical Sciences, Università di Napoli Federico II, Napoli, Italy; ^4^ Medical Oncology Unit, Istituto Nazionale per lo studio e la cura dei tumouri “Fondazione G. Pascale” IRCCS, Napoli, Italy; ^5^ Pathology Unit, Istituto Nazionale per lo Studio e la Cura dei Tumouri “Fondazione G. Pascale” IRCCS, Napoli, Italy; ^6^ Oncology Unit, AORN Cardarelli, Napoli, Italy; ^7^ Oncology Unit, Department of Clinical Medicine and Surgery, Università di Napoli Federico II, Napoli, Italy; ^8^ Department of Industrial Engeenering, Università di Napoli Federico II, Napoli, Italy; ^9^ Oncology Unit, Ospedale Civico, Lecce, Italy; ^10^ Oncology Unit, IRCCS Casa Sollievo della Sofferenza, San Giovanni Rotondo, Italy; ^11^ Endocrinology Unit, IRCCS Casa Sollievo della Sofferenza, San Giovanni Rotondo, Italy

**Keywords:** neuroendocrine tumours, Ki67 index, octreotide, lanreotide, somatostatin analogues

## Abstract

Somatostatin analogues (SSAs) have shown limited and variable antiproliferative effects in neuroendocrine tumours (NETs). Whether tumour control by SSAs depends on grading based on the 2010 WHO NET classification is still unclear. The aim of this study is to evaluate the efficacy of long-acting SSAs in NETs according to Ki67 index.

An observational Italian multicentre study was designed to collect data in patients with gastro-entero-pancreatic or thoracic NETs under SSA treatment. Both retrospective and prospective data were included and they were analysed in line with Ki67 index, immunohistochemically evaluated in tumour samples and graded according to WHO classification (G1 = Ki67 index 0-2%, G2 = Ki67 index 3-20%, G3 = Ki67 index > 20%).

Among 601 patients with NET, 140 with a histologically confirmed gastro-entero-pancreatic or thoracic NET or NET with unknown primary were treated with lanreotide autogel or octreotide LAR. An objective tumour response was observed in 11%, stability in 58% and progression in 31%. Objective response and tumour stability were not significantly different between G1 and G2 NETs. Progression free survival was longer but not significantly different in G1 than G2 NETs (median: 89 *vs* 43 months, *p* = 0.15). The median PFS was significantly longer in NETs showing Ki67 < 5% than in those showing Ki67 ≥5% (89 *vs* 35 months, *p* = 0.005).

SSA therapy shows significant antiproliferative effects in well differentiated low/intermediate-proliferating NETs, not only G1 but also in G2 type. A Ki67 index of 5% seems to work better than 3% to select the best candidates for SSA therapy.

## INTRODUCTION

Somatostatin analogues (SSAs) represent a consolidated therapeutic approach in patients with neuroendocrine tumour (NET). Octreotide and lanreotide have initially been shown effective in controlling endocrine syndromes associated with NETs [1, 2]. Subsequently, their role as antiproliferative agents has clearly been demonstrated in randomized trials conducted in patients with well differentiated NETs [3, 4]. Modern NET guidelines report that SSAs are currently a therapeutic option in patients with NETs, whatever the site of primary tumour, stage, or activity [5, 6, 7]. Moreover, SSAs are started early in the therapeutic algorithm, both for their efficacy in arresting tumour proliferation and for their manageability and excellent patients’ tolerance.

SSAs are more frequently shown to induce tumour stabilization (about 50-80% of patients with progressive well differentiated NETs) than objective responses (< 10%) [3, 4, 8, 9]. Recently, two randomized trials have been conducted in patients with NETs to evaluate the efficacy of long-acting slow-release SSA formulations and showed a significant antiproliferative effect [3, 4]. The PROMID study (double-blind placebo-controlled prospective randomized study), first demonstrated that octreotide LAR significantly prolonged the time to progression in a population of metastatic well-differentiated low-proliferating NETs of small intestine [3]. However, 95% of patients had tumours with Ki67 less than 2%. Subsequently, the CLARINET study (double-blind controlled study of Lanreotide anti-proliferative response in NET) performed in patients with non functioning well-/moderately differentiated GEP NETs (70% G1, 30% G2 with Ki67 ranging 3-10%) and including 45% pNETs, confirmed a significant improvement of progression free survival (PFS) in patients treated with lanreotide Autogel as compared to those receiving placebo [4].

These studies have changed the indication of SSA in NET therapy even if some concerns still require attention. In particular, it is unclear whether SSAs are effective in all type of well-/moderately differentiated NETs, regardless from Ki67 index.

Thus the aim of the current study was at analyzing the anti-tumour effects of long-acting lanreotide and octreotide in patients with NET according to Ki67 index.

## RESULTS

### PFS

**Figure 1:**
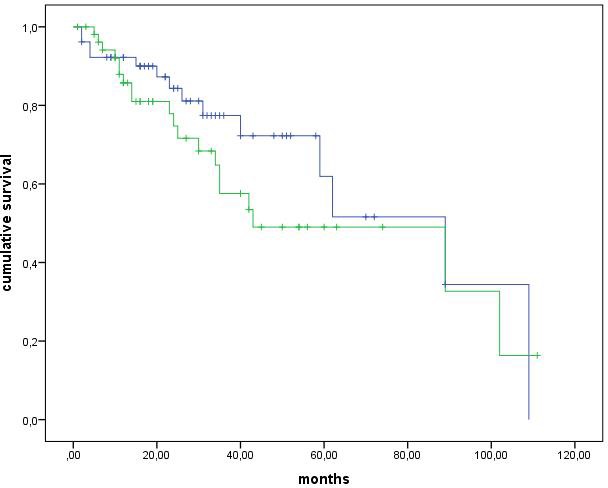
Log-rank analysis These Kaplan-Meier survival curves show progression free survival for patients with grading G1 and G2.

**Figure 2:**
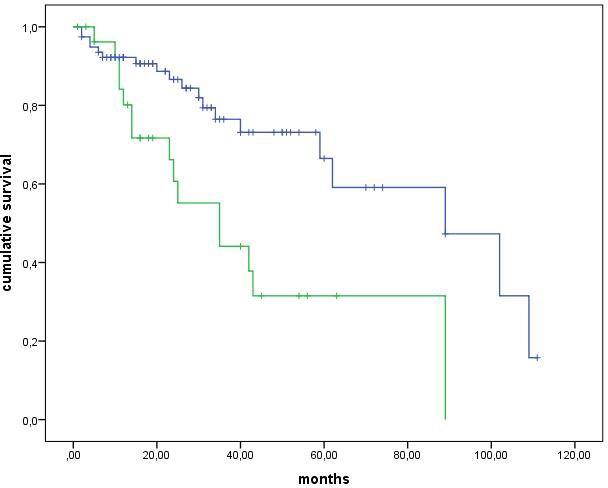
Log-rank analysis These Kaplan-Meier survival curves show progression free survival for patients with Ki67 < 5% and ≥5%.

The median PFS of 106 G1-G2 NET patients receiving SSA therapy was 89 months (CI interval, 58.9-119 months). PFS was higher but not significantly different in G1 than G2 NETs (median: 89 *vs*. 43 months, *p* = 0.15) (Figure 1). A Ki67 index of 5% was the best cut-off at the ROC analysis to separate patients according to tumour progression, with a sensitivity and specificity of 65 and 69%, respectively (*p* = 0.004). When this Ki67 cut-off was considered, PFS was significantly higher in NETs with Ki67 < 5% than in those with Ki67 ≥5% (median: 89 *vs*. 35 months, *p* = 0.005) (Figure 2). PFS was not different between GEP and thoracic NET (median: 89 *vs*. 59 months, *p* = 0.531), while was higher in GEP and thoracic NETs than in those with unknown primary tumour (median: 89 *vs.* 35 months, *p* = 0.048), in loco-regional than metastatic disease (median: 89 *vs.* 40 months, *p* = 0.005) and in. Within the GEP group, the median PFS was 62 months for pancreatic and 102 months for ileal NETs, without significant differences (*p* = 0.464). Within the thoracic group, the median PFS was 59 months for lung and 42 months for thymic NETs, without significant differences (*p* = 0.077). There was no difference between functioning and non-functioning tumours (median: 59 *vs.* 89 months, *p* = 0.710), as well as between sporadic and MEN1 (median: 59 *vs.* 89 months, *p* = 0.533) and between Octreoscan / ^68^Ga-PET positive and negative (median: 89 months *vs.* median not reached, *p* = 0.965). At the Cox regression analysis, both ki67 index ≥5% (Exp(B): 2.011, IC95%: 0.959-4.216) and distant metastases (Exp(B): 1.483, IC95%: 0.990-2.220) were independent negative prognostic factors.

### Tumour response

An objective tumour response was observed in 11% of cases. Tumour stability occurred in 58%, while tumour progression in 31% (Table 4). Objective response and tumour stability were not significantly different between G1 and G2 NETs, as well as between loco-regional disease and distant metastases (Table 4). Clinical benefit (including objective response and tumour stability) was not significantly different between G1 and G2 NETs, as well as between the group of patients with Ki67 index ≥5% and the one with Ki67 index < 5%. On the contrary, the clinical benefit was significantly higher in patients with loco-regional disease than in those with distant metastases (*p* = 0.002), as well as in patients with GEP NETs than others (*p* = 0.02). Tumour stage was not significantly different between the group of subjects with Ki67 index ≥5% and the one with Ki67 index < 5%.

Among the 14 patients who experienced a switch from standard to high dose SSA treatment, an objective response occurred in 2 patients (14.3%), while tumour progression in 2 others (14.3%). Stable disease was observed in the remaining 10 (71.4%). In 4 of 5 patients with clinical syndrome uncontrolled by standard dose of SSAs, a control of the syndrome was achieved on high dose regimen.

## DISCUSSION

NETs are well recognized to specifically express somatostatin receptors and to bind SSAs. Their antiproliferative effects on pancreatic and ileal NET cells were first demonstrated *in vitro* [12, 13, 14]. Since nineties, clinical studies reported SSAs to exert some anti-tumour efficacy in different types of NET. However, these data were based on not-randomized studies performed on small, heterogeneous and retrospective series of patients [8, 9, 15-18]. Published in 2009, the PROMID study has been a milestone in the therapy of NETs, because it was the first randomized prospective phase 3 trial investigating SSA therapy in NETs [3]. It was possible to demonstrate that SSAs are an effective and manageable therapeutic approach in well differentiated low-proliferating NETs of small intestine. The most relevant consequence was that SSAs were included in NET guidelines as a valid therapeutic option in patients with metastatic or inoperable well differentiated NETs [5-7]. The CLARINET study, an international randomized multicenter phase III trial, further enlarges the spectrum of NET types to consider candidates for SSA therapy [4]. In CLARINET, not only small intestine, but also pancreatic and hindgut NETs were included, showing a 53% risk reduction to tumour progression or death with lanreotide *versus* placebo. Besides, if PROMID was performed before the 2010 WHO classification of NETs and only included low-proliferating tumours with Ki67 < 2%, CLARINET was addressed not only to G1 but also to G2 with Ki67 < 10%, demonstrating that SSAs are effective in both.

If these two pivotal studies have promoted SSAs as one of the main systemic therapies of NETs, on the other hand some questions remain unsolved. In particular, it is not clear if all well differentiated NETs, regardless from Ki67 index, are responsive to SSAs. In this meaning, G2 with Ki67 between 10 and 20% and even those G3 with well differentiated histology and Ki67 < 50% could be potential candidates for SSA therapy. The current study aims to evaluate SSA therapy in NETs of different origin and grading and to establish the impact of Ki67 index in the response to SSA. Although the observational mainly retrospective study design represents a limitation of the study, some relevant results have been obtained. The most relevant finding was that PFS was not significantly different between G1 and G2, while it was significantly different according to Ki67 more or less than 5%. Median PFS was 89 months in NETs with Ki67 < 5% and 35 months in those with Ki67 ≥5%. This finding highlights a completely different clinical behaviour between these subgroups. It also explains why there is no difference in responsiveness to SSAs between G1 and G2 tumours which are separated by a Ki67 cut-off of 3%. At the opposite, a cut-off of 5% seems to well distinguish two subpopulations of well differentiated NETs, those with low proliferation rate and indolent clinical course and those with moderately high proliferation rate and tendency to progression. A previous study performed on 21 nonfunctioning pNETs highlighted that a Ki67 index < 5% correlated with tumour stability under treatment with octreotide LAR [19]. Again a Ki67 index of up to 5%, together with pretreatment stability and hepatic tumour load of up to 25%, were correlated with tumour stability under treatment with lanreotide [20]. A similar finding has been also reported in two prognostic studies performed in lung NETs, [21, 22] where a Ki67 cut-off of 4% was able to separate patients with significantly different disease free and overall survival.

Beyond these results, it is not negligible that SSAs are an effective therapeutic instrument also in well differentiated NETs with moderately high proliferation rate. In the current study, clinical benefit of SSAs was observed in 63% of G2 NETs, although objective response rate was really scarce and progression rate quite relevant in this subgroup. Even in G3 NETs is possible to hypothesize some efficacy of SSAs. Recently, a French study found a rate of 43% of well differentiated NETs in the G3 subgroup [23]. There was a significant difference between well and poorly differentiated G3 NETs in terms of octreoscan uptake (88 *vs* 50%) and median overall survival (41 *vs* 17 months). All responders to cisplatin chemotherapy were poorly differentiated G3 NETs. Of consequence, the well differentiated G3 NETs could constitute a NET subgroup different from other G3. SSAs could be effective in this subgroup as in G1-G2. In the current study, 30 patients of the 140 who were initially selected as SSA population belonged to the subgroup G3. All of them were excluded from this analysis because of concomitant systemic therapies. To avoid confounding results this subgroup will be analyzed in a separate study.

Interestingly, Ki67 index and tumour stage resulted to be both independent negative prognostic factors of progression at the multivariate analysis, suggesting that NET patients with Ki67 < 5% are expected to be the best responders to SSAs regardless from any other factor. This finding from a large series of NET patients is a helpful element to define the identikit of the best candidate to SSA therapy, following the initial indications pointed out by the PROMID and CLARINET studies [3, 4].

Another point of interest in NET therapy with SSA concerns the use of high dose schedules in place of standard dose [24]. To shorten the interval of administration of octreotide LAR from 30 mg every 28 days to 30 mg every 21 days was proven to slow tumour progression in patients with GEP and thoracic well differentiated NETs [25]. Previously, very high dose octreotide and lanreotide treatment resulted in high rate of tumour stabilization in patients with progressive well differentiated GEP NETs [26, 27]. In one study, where lanreotide was used in subcutaneous formulation at the dose of 15 mg a day, one complete and one partial objective response were also observed [26]. The current study was not focused on this aspect. However, in 14 of 106 patients there was a switch from standard to high dose SSA treatment, following tumour progression in 9 and uncontrolled clinical syndrome in 5. An objective response was observed in 14.3%, while stable disease in 71.4% of cases. Control of clinical syndrome was achieved in 80% of those patients who were still symptomatic under standard doses of SSAs.

In conclusion, SSA therapy shows significant antiproliferative effects in well differentiated low/intermediate-proliferating NETs, not only G1 but also in G2 type. A Ki67 index ≤5% seems to indicate the best candidates for SSA therapy. Further prospective studies need to be performed on this topic as well as on the role of SSA in G2 with Ki67 > 10% and in well differentiated G3 NETs, on the role of SSA high dose in NET therapy.

## PATIENTS AND METHODS

### Study design

This is an observational Italian multicentre study designed to collect data on patients with gastro-entero-pancreatic and thoracic NETs or NETs with unknown primary origin who were receiving treatment with SSA. The observational data have been collected through an e-CRF and stored in a centralized computer database ad hoc created. Both retrospective data of patients in treatment with SSA from 2005 and prospective data of patients treated with SSA from March 2012 to April 2014 were included. Objective response rate (ORR) and progression free survival were evaluated according to Ki67 index, evaluated by counting the percentage of nuclei positive to the Mib-1 primary antibody upon 2000 tumour cells.

Between January 2005 and April 2014, 601 patients diagnosed with NET in the five centres involved in the study were examined as potentially eligible for the study. Inclusion criteria were: a) a histologically documented diagnosis of NET, revised according to the last WHO classification criteria for NET of gastro-entero-pancreatic, bronchial and thymic origin [10, 11] b) Ki67 index immunohistochemically evaluated in representative tumour samples to grade tumours according to WHO classification of NETs (G1 = Ki67 index 0-2%, G2 = Ki67 index 3-20%, G3 = Ki67 index > 20%), c) treatment with SSAs at standard doses for at least 3 months (octreotide LAR 30 mg/28 d, lanreotide autogel 120 mg/28 d). In each center involved in the study, the pathological diagnosis was revised by at least two pathologists dedicated to NET and participating to the multidisciplinary tumour board for NET.

Exclusion criteria were: a) histology not revised according to the last WHO classifications, b) Ki67 index unavailable, c) treatment with SSAs for < 3 months. The study was conducted in accordance with the Declaration of Helsinki and approved by the Federico II University Ethics Committee (protocol n°227/2011 approved on 11/01/12). All patients gave written informed consent.

### Patient characteristics

Data of 140 patients fulfilling the inclusion criteria have been reported in the current study. Mean age was 59±2.6 yrs. Male:female ratio was 1.1 (Table 1). One-hundred twenty-three patients had a sporadic NET (88%) and 17 a MEN1-related NET (12%). The most frequent site was pancreas accounting for 44% of cases, followed by lung (19%) and ileum (12%) (Table 1). Tumour grading was G1 in 35%, G2 in 44%, G3 in 21%. Tumour stage included NETs without metastases in 26%, locoregional metastases in 29%, distant metastases in 45%. Primary tumour had been surgically removed in 66% of patients. NET was non-functioning in 85% of cases. Functioning NET accounted for 15% of cases, including Zollinger-Ellison syndrome (ZES) in 4.3%, hypoglycemic hyperinsulinemic syndrome in 3.6%, carcinoid syndrome in 7.1%. Abnormally increased neuroendocrine markers were serum chromogranin-A (CGA) in 47% patients, gastrin in 7.8%, insulin/C-peptide in 3.6% and/or 24-hour urinary 5-hydroxyindolacetic acid (5-HIAA) in 7.1% (Table 1).

**Table 1:** Patients' characteristics: 140 patients with G1-G2-G3 NET treated with somatostatin analogues

Parameters		n° of patients (%)
**Age**	mean±SE, range	59±2.6 (21-86)
**Gender M/F**		74 / 66
**Site of primary tumour**	Lung	26 (19)
	Thymus	5 (4)
	Stomach	8 (6)
	Pancreas	60 (44)
	Ileum	18 (12)
	Other sites*	7 (3)
	Unknown primary	16 (12)
**Biology**	Sporadic	123 (88)
	MEN1	17 (12)
**Grading**	G1	49 (35)
	G2	61 (44)
	G3	30 (21)
**Stage**	Loco-regional disease	62 (45)
	Distant metastases	78 (55)
**Nonfunctioning tumour**		119 (85)
**Functioning tumour**	Zollinger-Ellison syndrome	6 (4.3)
	Hypoglicemic syndrome	5 (3.6)
	Carcinoid syndrome	10 (7.1)
**Positive circulating NE markers**	Serum Chromogranin-A	66 (47)
	Serum Gastrin	11 (7.8)
	Serum Insulin/C-peptide	5 (3.6)
	24-h-urinary 5-HIAA	10 (7.1)
**Octreoscan**	Positive	46 (75)
	Negative	15 (25)
**^68^Ga-DOTATATE -PET**	Positive	29 (83)
	Negative	6 (17)

NET: neuroendocrine tumour; NE: neuroendocrine; 5-HIAA: 5-hydroxyindolacetic acid; *colon-rectum, duodenum, appendix.

A somatostatin receptor scintigraphy was performed in 61 patients, by using intravenous injection of Indium-111-DTPA-Phe1-octreotide (Octreoscan, Mallinckrodt Medical, Petten, The Netherlands; 120-200 MBq) with SPECT/CT fusion images. Octreoscan was positive in 46 cases (75%). A ^68^Ga-DOTATATE PET was performed in 35 patients, by administering 120-220 MBq of activity, with acquisition of images 45-60 min post-injection. There was a positive ^68^Ga-PET uptake in 29 cases (83%).

In 34 of the 140 cases (24%), including 30 with G3 (100% of this subgroup) and 4 with G2 NET (6% of this subgroup), other systemic anti-tumour therapies were started concomitantly or within 3 months from the beginning of SSA therapy. In particular, other therapies concomitant to SSA were chemotherapy in 27 cases and targeted therapy in 7 cases. To limit confounding data, we excluded from final analysis all patients with G3 and 4 patients with G2 NET treated with therapies other than SSAs. Finally 106 patients, entering all the inclusion criteria and resulting to be affected with G1 (49 patients) and G2 NET (57 patients), according to Ki67 index, form the basis for the statistical analysis (Table 2). Both in G1 and G2 NETs, pancreas was the main primary site followed by ileum and lung. Sporadic NETs accounted for the most of cases while MEN1 NETs were about one fourth of G1 tumours and a minority of G2. Tumour stage was equally balanced between G1 and G2. NET was functioning in 79% of G1 and 88% of G2, respectively. The most frequent syndrome was carcinoid syndrome in G1 and ZES in G2. Abnormally increased serum CGA occurred in 55% of G1 and 60% of G2. Octreoscan and ^68^Ga-DOTATATE PET uptake was similar in G1 and G2 (Table 2).

**Table 2:** Patients' characteristics: 106 patients with G1-G2 NET treated with somatostatin analogues

Parameters		G1 *n*° (%)	G2 *n*° (%)
**Total number**		49 (46)	57 (54)
**Age**	mean±SE, range	56+16	60+12
**Gender M / F**		27 / 22	27 / 30
**Site of primary tumour**	Lung	6 (12)	7 (12)
	Thymus	1 (2.0)	4 (7.0)
	Stomach	3 (6.1)	5 (8.8)
	Pancreas	29 (59)	20 (35)
	Ileum	5 (10)	10 (17)
	Other sites*	1 (2.0)	6 (10)
	Unknown primary	4 (8.2)	5 (8.8)
**Biology**	Sporadic	36 (73)	53 (93)
	MEN1	13 (27)	4 (7.0)
**Stage**	Loco-regional disease	24 (49)	27 (47)
	Distant metastases	25 (51)	30 (53)
**Nonfunctioning tumour**		40 (79)	49 (88)
**Functioning tumour**	Zollinger-Ellison syndrome	2 (4.1)	3 (6)
	Hypoglicemic syndrome	3 (6)	1 (2)
	Carcinoid syndrome	4 (11)	4 (4)
**Positive circulating NE markers**	Serum Chromogranin-A	27 (55)	34 (60)
	Serum Gastrin	10 (20)	11 (19)
	Serum Insulin	2 (4.1)	2 (3.5)
	24-h-urinary 5-HIAA	4 (8.2)	3 (5.3)
**Octreoscan**	Positive	20 (80%)	19 (76%)
	Negative	5 (20%)	6 (24%)
**^68^Ga-DOTATATE -PET**	Positive	14 (93%)	14 (93%)
	Negative	1 (7%)	1 (7%)

NET: neuroendocrine tumour; NE: neuroendocrine; 5-HIAA: 5-hydroxyindolacetic acid; *colon-rectum, duodenum, appendix

### Treatment with SSA

Initial SSA treatment included octreotide LAR 30 mg/28 d in 71 patients and lanreotide autogel 120 mg/28 d in 35 patients. The median treatment duration with SSA was 23 months (range, 3-88 months). The initial SSA dose subsequently changed in 14 patients, because of tumour progression in 9 and uncontrolled clinical syndrome in 5. Octreotide was switched to lanreotide in 6 cases (Table 3). At the final follow-up under SSA treatment, 65 patients received octreotide LAR and 41 patients received lanreotide autogel.

**Table 3:** Somatostatin analogue schedule treatment in 106 patients with G1-G2 NET

	Schedule	Number of Patient
**Initial treatment**	LAN 120 mg/28 d	35
	LAR 30 mg/28 d	71
**First treatment switch**	LAN from 120 mg/28 d to LAN 120 mg/21 d	2
	LAR from 30 mg/28 d to LAR 30 mg/21 d	6
	LAR from 30 mg/28 d to LAN 120 mg/28 d	2
	LAR from 30 mg/28 d to LAN 120 mg/21 d	2
	LAR from 30 mg/28 d to LAN 90 mg/21 d	2
**Second treatment switch**	LAN from 120 mg/28 d to LAN 120 mg/21 d	2
	LAN from 120 mg/21 d to LAN 120 mg/14 d	4
	LAN from 90 mg/21 d to LAN 120 mg/21 d	2
	LAR from 30 mg/21 d to LAR 30 mg/14 d	2
**Final treatment**	LAN 120 mg/28 d	33
	LAN 120 mg/21 d	4
	LAN 120 mg/14 d	4
	LAR 30 mg/28 d	59
	LAR 30 mg/21 d	4
	LAR 30 mg/14 d	2

**Table 4:** Rate of tumour response to treatment with somatostatin analogues in 106 pts with G1-G2 NET

	**Tumour Response**
**Total patients**	n° (%)
Complete Response	2 (2.0)
Partial Response	10 (9.0)
Stable Disease	61 (58)
Progression	33 (31)
**G1 NET**	
Complete Response	1 (2.0)
Partial Response	3 (6.0)
Stable Disease	33 (67)
Progression	12 (25)
**G2 NET**	
Complete Response	1 (2.0)
Partial Response	7 (12)
Stable Disease	28 (49)
Progression	21 (37)
**Patients with Lung – Thymus NET**	
Complete Response	0
Partial Response	1 (6.0)
Stable Disease	11 (61)
Progression	6 (33)
**Patients with Pancreas NET**	
Complete Response	0
Partial Response	6 (12)
Stable Disease	30 (61)
Progression	13 (27)
**Patients with Gastro-Intestinal NET**	
Complete Response	2 (7.0)
Partial Response	2 (7.0)
Stable Disease	16 (53)
Progression	10 (33)
**Patients with unknown primary NET**	
Complete Response	0
Partial Response	1 (11)
Stable Disease	4 (44)
Progression	4 (44)
**Patients with loco-regional disease**	
Complete Response	1 (2.0)
Partial Response	5 (10)
Stable Disease	37 (72)
Progression	8 (16)
**Patients with distant metastases**	
Complete Response	1 (1.8)
Partial Response	5 (9.2)
Stable Disease	24 (44)
Progression	25 (45)

NET: neuroendocrine tumour

### Efficacy and safety assessment

Efficacy was evaluated in terms of progression free survival (PFS) and tumour response according to the RECIST definitions for tumour [19]. The radiologic assessment of tumour lesions was performed by contrast enhanced computerized tomography (CT) or magnetic resonance imaging (MRI), at baseline and every 3-6 months during the follow-up period. Endoscopy and endoscopic ultrasonography (EUS) were also performed in combination with CT/MRI where appropriate. In patients experiencing increase of SSA dose after initial tumour progression, PFS was evaluated at the last SSA dose.

### Statistical analysis

The statistical analysis was performed with the SPSS package (Cary, NC, USA). Data were expressed as mean±SEM. The significance was set at 5%. Comparisons of tumour responses among different groups of patients were performed with the Chi-square test with Yates correction or Fisher exact test. PFS was calculated using the Kaplan-Meier method and comparison between subgroups was performed by using the log-rank test. Cox regression analysis was performed to compare variables which were significant at the univariate test. The Ki67 index was analyzed by receiver operator characteristic (ROC) analysis by using a non parametric model to determine the best cutoff to distinguish patients who are in progression from those who are not in progression.
